# A normothermic *ex vivo* organ perfusion delivery method for cardiac transplantation gene therapy

**DOI:** 10.1038/s41598-019-43737-y

**Published:** 2019-05-29

**Authors:** Muath Bishawi, Jun-Neng Roan, Carmelo A. Milano, Mani A Daneshmand, Jacob N. Schroder, Yuting Chiang, Franklin H. Lee, Zachary D. Brown, Adam Nevo, Michael J. Watson, Trevelyn Rowell, Sally Paul, Paul Lezberg, Richard Walczak, Dawn E. Bowles

**Affiliations:** 1 Division of Cardiothoracic Surgery, Department of Surgery, Duke University, Durham, NC USA; 2 Department of Biomedical Engineering, Pratt School of Engineering, Duke University, Durham, NC USA; 3 Division of Cardiovascular Surgery, Department of Surgery, College of Medicine, National Cheng Kung University Hospital, Tainan City, Taiwan; 4 Perfusion Services, Duke University, Durham, NC USA; 5 TransMedics, Inc., Andover, MA USA; 6 Division of Surgical Sciences, Department of Surgery, Duke University, Durham, NC USA

**Keywords:** Cardiovascular biology, Cardiac device therapy

## Abstract

Clinically, both percutaneous and surgical approaches to deliver viral vectors to the heart either have resulted in therapeutically inadequate levels of transgene expression or have raised safety concerns associated with extra-cardiac delivery. Recent developments in the field of normothermic *ex vivo* cardiac perfusion storage have now created opportunities to overcome these limitations and safety concerns of cardiac gene therapy. This study examined the feasibility of *ex vivo* perfusion as an approach to deliver a viral vector to a donor heart during storage and the resulting bio distribution and expression levels of the transgene in the recipient post-transplant. The influence of components (proprietary solution, donor blood, and *ex vivo* circuitry tubing and oxygenators) of the Organ Care System (OC) (TransMedics, Inc., Andover MA) on viral vector transduction was examined using a cell-based luciferase assay. Our *ex vivo* perfusion strategy, optimized for efficient Adenoviral vector transduction, was utilized to deliver 5 × 10^13^ total viral particles of an Adenoviral firefly luciferase vector with a cytomegalovirus (CMV) promotor to porcine donor hearts prior to heterotopic implantation. We have evaluated the overall levels of expression, protein activity, as well as the bio distribution of the firefly luciferase protein in a series of three heart transplants at a five-day post-transplant endpoint. The perfusion solution and the *ex vivo* circuitry did not influence viral vector transduction, but the serum or plasma fractions of the donor blood significantly inhibited viral vector transduction. Thus, subsequent gene delivery experiments to the explanted porcine heart utilized an autologous blood recovery approach to remove undesired plasma or serum components of the donor blood prior to its placement into the circuit. Enzymatic assessment of luciferase activity in tissues (native heart, allograft, liver etc.) obtained post-transplant day five revealed wide-spread and robust luciferase activity in all regions of the allograft (right and left atria, right and left ventricles, coronary arteries) compared to the native recipient heart. Importantly, luciferase activity in recipient heart, liver, lung, spleen, or psoas muscle was within background levels. Similar to luciferase activity, the luciferase protein expression in the allograft appeared uniform and robust across all areas of the myocardium as well as in the coronary arteries. Importantly, despite high copy number of vector genomic DNA in transplanted heart tissue, there was no evidence of vector DNA in either the recipient’s native heart or liver. Overall we demonstrate a simple protocol to achieve substantial, global gene delivery and expression isolated to the cardiac allograft. This introduces a novel method of viral vector delivery that opens the opportunity for biological modification of the allograft prior to implantation that may improve post-transplant outcomes.

## Introduction

Cardiovascular diseases (CVD) remain the leading cause of death worldwide^1^. The number of CVD patients with heart failure (HF) in the US is approaching 6.5 million adults and is estimated to increase by 46% in the next decade so that there will be more than 8 million adults in the US with HF by 2030^1^. HF has no cure and about 50% of people who develop HF die within five years of diagnosis. Once a patient develops end stage heart failure, therapeutic options are limited to palliative care, some type of mechanical circulatory support or cardiac transplantation. While cardiac transplantation remains the gold standard therapy for qualifying patients, it is still limited by the supply of organs, and fraught with post-transplant complications such as graft dysfunction, allograft vasculopathy, rejection, and the side effects of immunosuppression^2^. There continues to be a need for improvement in cardiac transplantation and gene therapy approaches may be able to address some of these complications.

A successful gene therapy approach based on viral vectors requires three elements: delivery vehicle (a capsid shell), a therapeutic target (a transgene) and a physical method of delivery into the tissue(s) of interest (direction injection, intravenous (IV) administration etc.)^3^. Proof of concept of the benefit of gene therapy in the context of heart transplant to ameliorate deleterious responses to the graft in the recipient has been demonstrated in rodent heart transplant models using cold static storage or Langendorff delivery methods^4,5^. Of the two delivery approaches, it is unlikely that standard of care organ storage (cold static storage) will facilitate translation of gene therapy for transplantation since many aspects of the viral vector transduction process such as receptor entry, uptake, trafficking through the cell, nuclear import, and efficient disassembly are temperature and metabolism dependent^6^. Isolated perfusion systems such as a Langendorff have been utilized experimentally for over 90 years in physiological and pharmacological research to evaluate cardiac function *ex vivo*^7^. These perfusion strategies maintain normothermic and aerobic metabolism, facilitating the biochemical and molecular steps necessary for viral uptake into the heart.

Several companies have developed *ex vivo* perfusion systems intended to mitigate ischemic injury during organ preservation. Clinically, these devices may replace the cold static storage preservation strategy for solid organ transplant. An *Ex vivo* warm blood perfusion system (The Organ Care System (OCS) TransMedics Inc. Andover MA) has been the most clinically tested device for cardiac transplantation^8^. This device is portable and is primed with heparinized donor blood mixed with a proprietary perfusion solution. Once on the device, the heart is maintained in a nonworking but metabolically active mode. This device has achieved successful clinical *ex vivo* perfusion for prolonged periods of time^9^. While the main goal of perfusion storage is to reduce ischemia reperfusion injury, improve the safety, and extend the time of the preservation phase, perfusion storage uniquely isolates the metabolically active cardiac graft, potentially enabling biological modification. Concerning possible gene therapy, this type of perfusion storage allows for intracoronary delivery of high concentrations of viral vectors with continuous recirculation under metabolically favorable conditions. The aim of this study was to evaluate the utility of *ex vivo* warm blood perfusion as a method of viral vector delivery to the heart in a porcine transplant model.

## Methods

### Animals

Outbred Yorkshire pigs (females of approximate weight of 30–35 kg) were used in this study. All work in this report has been approved by Duke University Institutional Animal Care and Use Committee. All experiments were performed in accordance with relevant guidelines and regulations. Transplant and recipient pig were littermates of compatible blood types.

### Recombinant adenoviral vector

The adenoviral (Ad) luciferase vector (serotype 5) was obtained from the Pittsburgh Human Gene Therapy Center (Pittsburgh, PA) and was used previously in our laboratory^10^.

### Cell based luminometer assays

Luminometry (either cell or tissue-based) was performed with a Veritas luminometer (Turner Biosystems, Sunnyvale, CA). HeLa cells were plated at 10,000 cells per well in 96-well plates. The cells were infected with 1000 particles/cell of Ad-CMV luciferase in the presence of normal growth media (DMEM, 10% FBS) and additional test additives including OCS solution, whole blood, plasma, and serum. 24 hours post infection, the 96-well plates were processed and light emission per well was determined as described previously in Messina *et al*.^11^.

### Viral vector delivery and heart transplantation

The donor heart was procured in a standard fashion with modifications described below specific to OCS perfusion. Heparin was administered (300 U/kg/IV), and approximately 1–1.3 liter of blood was drained directly from the right atrium prior to cross clamp. After cross clamping the ascending aorta, 500 ml cold del Nido cardioplegia (plasmalyte A, pH 7.4 (994 ml); Mannitol, 25% (13 ml); Magnesium Sulfate, 50% (4 ml); Sodium bicarbonate, 1 mEq ml, (13 ml); posassium chloride, 2 mEq/ml (13 ml); sterile water for injection (3 ml); Lidocaine HCL 2% (6.5 ml); mixed at Duke Compounding Facility) was delivered into the aortic root to arrest the heart. The heart was excised and prepared for the OCS device. The superior and inferior vena cavae were over-sewn; the ascending aorta was cannulated to serve as perfusion inflow, while the main pulmonary artery was cannulated to collect the heart’s venous drainage. The pulmonary veins and left atrium were left open and a vent was placed through one of the veins, across the mitral valve in the LV. Ventricular pacing leads were placed to maintain a rate of at least 80 beats per minute. Concurrently, the 1–1.3 liters of donor pig blood which was acquired at the time of organ harvest was diluted 1:1 with Plasma Lyte A (Baxter HealthCare Corporation) in reservoir and washed with 1 L physiological solution using a 250 ml Brat 2 bowl and a CellSaver (Brat 2) Autologous Blood Recovery System (Haemonetics, Braintree, MA).

Preparation of the perfusion solution deviated from the standard OCS protocol in three aspects: (a) blood washing described above, (b) adjustment of blood cell/OCS solution mixture and (c) addition of 5 × 10^13^ total viral particles of Adenoviral CMV-luciferase vector (Ad CMV-luc). First, the washed red blood cell fraction was reconstituted with the components shown in Table 1 then 5 × 10^13^ Ad CMV-luc was added to the mixture. Following a 5–15 minute priming of the circuit with this mixture (same time needed for preparing the heart for the OCS ~20 minutes), the heart was added to the device and maintained for 2 hours. Target mean perfusion pressure was 65–70 mmHg, and target coronary flow rate was 600 ml per minute. Samples of the blood/OCS/viral vector mixture were acquired during the initial set-up, pump priming, and at 15–30 minutes intervals during the perfusion run for study.

**Table 1:** Composition of perfusate for OCS machine.

Component	Concentration/ML of Total Circuit
**Blood Collection and Autotransfusion −750 ML**	
Post autotransfusion yield	**n/a**
**Blood Reconstitution 260 ML**	
Plasmalyte 200 ml	**n/a**
Albumin	7.7 mg/ml
Heparin	6.15 iu/ml
**Circuit Prime 604 ML**	
Transmedics priming solution 500 ml	n/a
Albumin	7.7 mg/ml
Ciprofloxacin	0.06 mg/ml
Cefazolin	0.62 mg/ml
Adult Multi-V	1 unit
Solumedrol	0.15 mg/ml
Sodium Bicarbonate	0.012 mEq/ml
**Corrective Medications 12 ML**	
Calcium Gluconate	0.37 mg/ml
Dextrose	0.615 mg/ml
Sodium Bicarbonate	0.003 mEq/ml

Final volume of the circuit was 1626 ml.

After 2 hours of perfusion on the OCS device, the heart was re-arrested with del-Nido crystalloid solution. The heart was then implanted in a blood type compatible recipient animal in a heterotopic fashion^12^. The pulmonary artery from the allograft was anastomosed in an end to side fashion to the infra-renal IVC, and the ascending aorta from the allograft anastomosed in a similar fashion to the infra-renal abdominal aorta. Recipient pigs were pre-treated with a 1000 mg solumedrol bolus followed by maintenance immunosuppression with prednisolone, cyclosporine, and imuran as described by Swindle *et al*.^13^. The animals were assessed daily for vital signs and graft function via palpation of the beating heart and with echocardiography.

The allograft, native heart, and samples from other organs (liver, lungs, spleen, psoas muscle) were procured on day 5 post-transplant at the time of euthanasia. Prior to harvest, graft function was evaluated by echocardiography. The abdominal aorta and thoracic aorta were both cannulated, and both hearts were arrested simultaneously using del-Nido solution infusion. The tissues were explanted, sectioned, and flash frozen in liquid nitrogen for assessment of transgene DNA, enzymatic activity and protein expression.

### Assessment of transgene activity and expression

#### Luciferase assay

Tissue samples (500 mg) were pulverized using a mortar and pestle and incubated for 30 minutes in 500 ul of passive lysis buffer (Promega, Madison, WI), then centrifuged for 15 min at 1300 rpm. Protein concentration of the resulting supernatant was determined using the Pierce BCA protein assay kit and a biokinetics reader (EL-340; BioTek Instruments). Equivalent protein amounts of the supernatant were assessed for luciferase activity using the Luciferase Assay Reagent (ONE-Glo, Promega, WI) per the manufacturer’s instruction. The light emission was measured using a Veritas luminometer (Turner Biosystems, Sunnyvale, CA).

#### Western blotting

Flash frozen sections of tissue were homogenized at 4 °C in lysis buffer (0.1% Triton X-100, 25 mM Tris-HCl, 150 mM NaCl, pH 7.4, 5 mM EDTA, Pierce Protease Inhibitor Minitablet (Pierce, product# 88665). Homogenates were assayed for protein concentration (BCA assay, Pierce) and equivalent amounts of protein were added to the gels (Tris-Glycine, 4–12% gradient, Invitrogen). Blots were blocked (5% nonfat milk in Tris buffered saline with Tween 20 (TBST)). Blots were then incubated with primary antibody (rabbit anti-firefly luciferase, Abcam product# ab21176). After washing, an anti-rabbit IgG secondary antibody conjugated to horse radish peroxidase was added (Invitrogen SA1-200). Blots were them developed using Enhanced Chemiluminescent Substrate (ECL) (ThermoFisher Scientific Pierce).

#### Immunostaining

Immunostaining of tissue sections was done using a primary Rabbit Anti-Firefly Luciferase antibody (Abcam product # ab21176) and a Donkey Anti-Rabbit IgG secondary conjugated to Alexa Fluor 594 (Abcam product # ab150076). 15-micron sections of the tissue were placed on slides and kept at −80 °C. The slides were then washed using TBS plus 0.05% Triton X-100 buffer. Samples were blocked in 10% goat serum with 1% BSA. Primary antibody (12 hours at 4 °C) was used per manufacture’s recommended dilution. After washing, the secondary antibody was added at the manufacture’s recommended concentration for 1 hour at room temperature. Fluorescent imaging was done using a Leica SP5 confocal system.

#### Quantitative real time PCR (qPCR) analysis

qPCR was used to determine viral genome copies in allograft and control tissues. Tissue samples were acquired at the time of animal sacrifice and stored in liquid nitrogen until DNA isolation. Total DNA was isolated with a DNeasy Blood and Tissue Kit (Qiagen). DNA purity and concentration were assessed using a NanoDrop Spectrophotometer. qPCR was performed for the Luciferase gene using the iQ SYBR Green Supermix (Bio-Rad) and the CFX Connect Real-Time PCR Detection System (Bio-Rad, Hercules, CA) with 30 cycle amplification of 95C for 10 seconds; 59.5C for 10 seconds; 72C for 30 seconds. A standard curve was generated using known concentrations of the CMV-Luc plasmid via serial 1:10 dilutions. Starting luciferase gene copy number was estimated for each of the samples and reported as viral copies per starting amount of total DNA isolated. The primers used for Luciferase template amplification were (Forward-5′-CTCACTGAGACTACATCAGC-3, and Reverse-5′-TCCAGATCCACAACCTTCGC-3).

## Results

### Evaluation of *ex vivo* perfusion components on viral vector transduction

**Figure 1:**
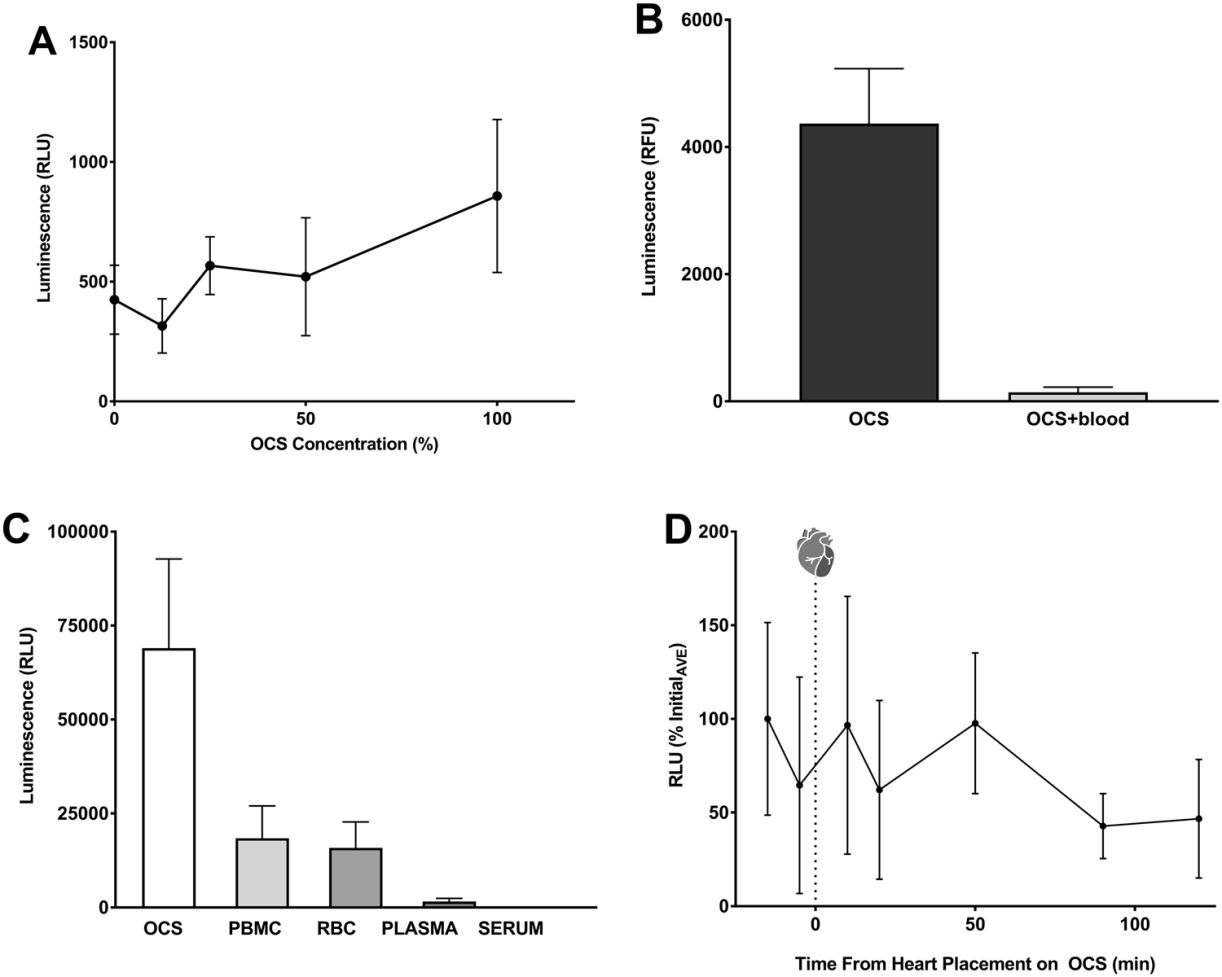
Cell based assessment of OCS components on viral vector transduction efficiency. (**A**) Influence of OCS solution. 1000 particles Ad luciferase per Hela cell were mixed with media and increasing percentage of OCS solution and used to infect Hela cells. RLU were determined 24 hours post infection. Data is shown fold change in RLU compared to untransduced Hela cells. (**B**) Influence of whole blood. Same as 1A except OCS solution was mixed with whole porcine blood. (**C**) Influence of blood components. Cell based luminescence assay to measure Ad Luciferase transduction efficiency with OCS solution and pig blood components. (**D**) Influence of OCS circuitry. Viral transduction efficiency over time while on the circuit with a heart. OCS circuit was set up with washed donor pig blood, Ad Luciferase and the donor heart. (RLU = relative light units).

Our study examined the feasibility of using normothermic *ex vivo* perfusion as a delivery system to administer biologicals (such as viral vectors) to the donor heart prior to transplantation. There are many components of the *ex vivo* perfusion system and each of these may inhibit the viral transduction process. In order to evaluate the influence of the major components of the OCS on the transduction efficiency of an Adenoviral-luciferase serotype 5 vector we utilized a cell -based luciferase assay to assess the influence of biologicals and chemicals on viral vector transduction^11^. As can be seen in Fig. 1A, the perfusion solution did not interfere with the ability of the Ad luciferase vector to transduce HeLa cells at any concentration of solution tested. However, when the transduction experiments were performed in the presence of a perfusion solution/whole blood (porcine) mixture, an almost complete reduction in transduction was observed (Fig. 1B). In order to determine which of the porcine blood components interfered with Ad vector transduction in the context of the perfusion solution, blood derivatives (Red Blood Cells (RBC), Peripheral Blood Mononuclear Cells (PBMC), serum, and plasma) were individually assessed in the cell-based assay. All of the blood components tested inhibited Ad transduction to some degree (Fig. 1C) with both the plasma and serum almost completely inhibiting transduction. The inhibitory influence of the plasma or serum was minimized when a donor heart was placed upon the circuit with a perfusate consisting of perfusion solution reconstituted with blood cells, which had been obtained following blood washing using a Cell Saver Device (Table 1). Ad vector was added to the perfusion system and re-circulated for 2 hours. Perfusate from this experiment was obtained at various time points before and after the addition of the donor heart and perfusate samples were evaluated for transduction in the cell-based assay. As can be seen in Fig. 1D, the ability of the viral vector to transduce Hela cells was not appreciably affected at any time point in the presence of the fully assembled *ex vivo* perfusion device. Statistically insignificant reduction in transduction efficiency at the end of the perfusion period may represent the vector uptake onto components of the circuit or into the heart.

### Gene delivery of adenoviral vector during *ex vivo* normothermic perfusion and heterotopic heart transplant

**Figure 2:**
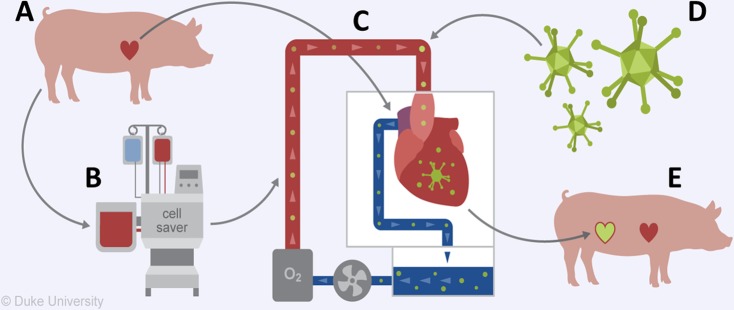
Experimental Overview. (**A**) Organ and blood donation. (**B**) Washing of donor blood, C. *ex vivo* perfusion using washed donor blood. (**D**) Addition of viral vector followed by. (**E**) Heterotopic heart transplant. “Illustrated by Lauren Halligan, MSMI; copyright Duke University; with permission under a CC BY-ND4.0 license”: https://creativecommons.org/licenses/by-nd/4.0/legalcode.

The overall experimental strategy of this approach is depicted in Fig. 2. First, the donor pig provided both the donor heart and blood volume for the OCS circuit (Fig. 2A). The whole blood from the donor pig was washed using a cell saver/autotransfusion approach to isolate the red blood cells from the serum elements (Fig. 2B) and then reconstituted using components to match the osmotic pressures of whole blood. (Table 1) The washed and reconstituted blood was mixed with OCS solution and used to prime the *ex vivo* circuit (Fig. 2C). Then 5 × 10^13^ particles of Ad-CMV luciferase were added directly to the circuit (Fig. 2D). The heart was then placed on the *ex vivo* perfusion device and perfused for 2 hours after which it was transplanted into the abdomen of the recipient animal (Fig. 2E).

### Evaluation of transgene expression post-transplant

**Figure 3:**
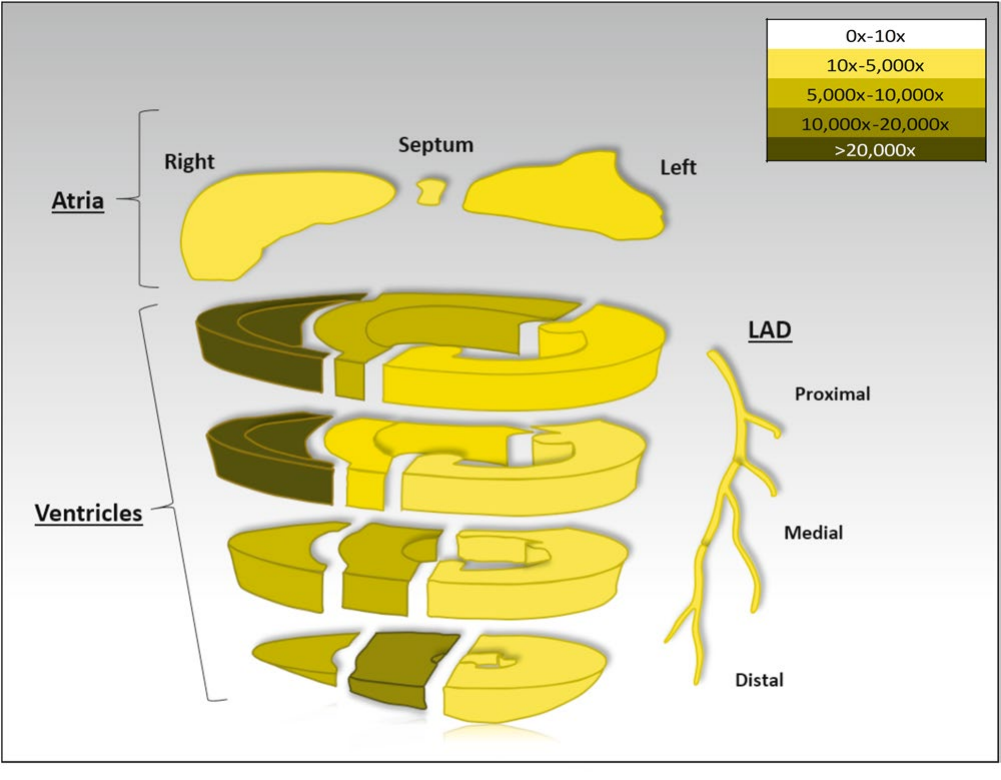
Luciferase protein activity. Fold change in Luciferase activity in allograft compared to native heart by different heart regions. N = 3 transplant experiments.

Three separate transplant experiments were conducted successfully with no adverse events seen in the recipient animals. All allografts were viable at five days post-transplant but were not fully interrogated for rejection by histological examination. We have evaluated the overall transgene DNA copy number, levels of protein expression, protein activity, as well as the bio distribution of the firefly luciferase protein in these three heart transplants 5 days post-transplant. The allograft and native heart were excised from the animals and regions of the heart (RV, LV, ventricular septum) were subdivided into sections. Each section was assessed for luciferase enzyme activity and summary data of all three hearts are shown in Fig. 3 and Table 2. The luciferase protein activity in the donor heart appeared robust across all areas of the myocardium as well as in the coronary arteries. At a minimum luciferase activity was 10 times that of the recipient’s native heart, and at a maximum, it was 20,000 fold higher than the native heart (Fig. 3). While all areas of the heart displayed high luciferase activity, this luciferase activity was not evenly distributed.

**Table 2:** Luciferase expression levels from naïve heart, three native hearts, and three allografts.

*Heart Region*	*Naïve heart RLU*/*mg protein*	*Native heart* (*n* = 3) *RLU*/*mg protein*	*Allograft 1 RLU*/*mg protein*	*Allograft 2 RLU*/*mg protein*	*Allograft 3 RLU*/*mg protein*
**Left ventricle sections**					
Atrioventricular junction	104.4 ± 21.4	67.4 ± 49.9	1630.0 ± 368.4	186132.8 ± 4412.1	91204.0 ± 18571
Middle	104.4 ± 30.0	60.7 ± 36.8	3908.0 ± 382.8	145482.6 ± 782.1	659463.0 ± 9544.59
Juxta apex	131.1 ± 36.7	287.4 ± 185.1	28053.0 ± 594.2	643349.4 ± 6362.2	667541.2 ± 5044.4
Apex	115.5 ± 45.3	747.3 ± 853.2	14560.1 ± 1146.1	788750.4 ± 33528.2	1437765.5 ± 8713.1
**Right ventricle sections**					
Atrioventricular junction	771.1 ± 1049.7	80.5 ± 107.6	12226.9 ± 1065.4	12708147.5 ± 283221.4	316520.9 ± 18900.4
Middle	71.1 ± 33.5	43.3 ± 23.0	2293.4 ± 280.9	740159.9 ± 26207.5	2366458.6 ± 22373.2
Juxta apex	126.6 ± 33.3	81.3 ± 18.0	27987.2 ± 4801.6	2672829.0 ± 33050.2	392790.5 ± 6131.9
Apex	137.7 ± 36.7	456.7 ± 618.4	1994.4 ± 132.1	782733.2 ± 6536.0	9099456.5 ± 73751.5
**Ventriclular septum sections**					
Atrioventricular junction	186.6 ± 103.7	66.6 ± 51.5	13835.0 ± 749.4	635594.0 ± 13516.8	745212.5 ± 17625.5
Middle	122.2 ± 7.6	44.6 ± 51.1	8495.7 ± 278.6	163896.0 ± 2359.3	449768.9 ± 9206.1
Juxta apex	111.1 ± 10.1	187.6 ± 245.7	36349.7 ± 1094.9	2898714.2 ± 94065.9	1668397.2 ± 21115.1
Apex	148.8 ± 30.0	62.3 ± 61.2	2998.3 ± 435.3	93257.7 ± 2506.8	2434775.2 ± 58712.1

Data were presented as mean ± SD. RLU = relative light units.

**Figure 4:**
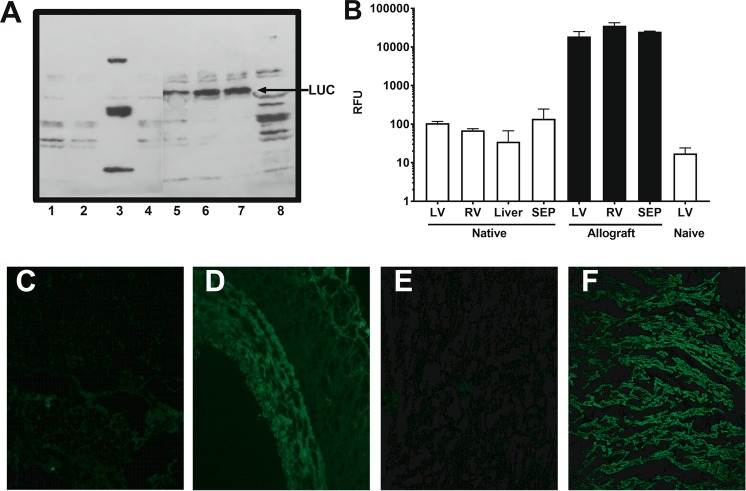
Luciferase protein expression in transplanted heart. (**A**) Lane1 Native LV, Lane 2 Native RV, Lane 3 Liver, Lane 4 native Septum, Lane 5 allograft LV, Lane 6 allograft RV, Lane 7 allograft septum, Lane 8. Naïve pig LV. (**B**) Luciferase activity from tissues corresponding to Fig. 4a lanes 1–8. (**C–F**) Immunostaining for Luciferase protein. (**C**) Native coronary, (**D**). Allograft coronary, (**E**) native LV, (**F**). Allograft LV. (Note: (**A**), the gel represents two different gels. For the image, these two gels were combined between lanes 4 and 5 and an additional ladder and a positive control lane with HeLa cells transfected with Ad-Luc lane removed for better display).

In addition to the quantitation of luciferase activity, protein lysates from all animals were examined for evidence of luciferase protein (Fig. 4A) in regions of the heart and in various organs. As can be seen in Fig. 4A, the firefly luciferase protein of 62 kDa was present at high levels in lysates from the LV, RV, and interventricular septum of the allograft (Lanes 5–7) but was not observed in native heart LV, RV, or septum (lanes 1, 2, or 4). A protein of 62 kDa corresponding to the luciferase protein was not observed in LV lysates from a naïve pig i.e. a control pig that did not receive a transplant and that had not been administered the Ad viral vector (lane 8). Luciferase activity corresponding to each of the tissues in Fig. 4A is shown in Fig. 4B. Importantly, Luciferase activity was only evident in tissues that expressed the 62 kDa protein (allograft tissues only). Luciferase activity and luciferase protein was not observed in native tissues or naïve tissue.

Transgene expression at the protein level was further verified using immunostaining. Figure 4 c, d demonstrates high abundance of the luciferase protein in virtually all myocardium examined with no staining seen in the control native myocardium. Furthermore, staining of the LAD demonstrated excellent luciferase staining in all layers of the artery (Fig. 4E,F).

Luciferase activity in all other organs of the recipient was similar to background suggesting minimal washout of the vector to remote tissue (Table 3).

**Table 3:** Luciferase activity levels measured in organs from recipient.

Tissue	RLU/MG Protein
Liver	6.5 ± 3.0
Lung	6.4 ± 6.9
Spleen	11.2 ± 9.7
Psoas muscle	7.9 ± 2.0
Aorta adjacent to graft	10.2 ± 11.1
IVC adjacent to graft	20.9 ± 12.6

RLU = relative light units.

**Figure 5:**
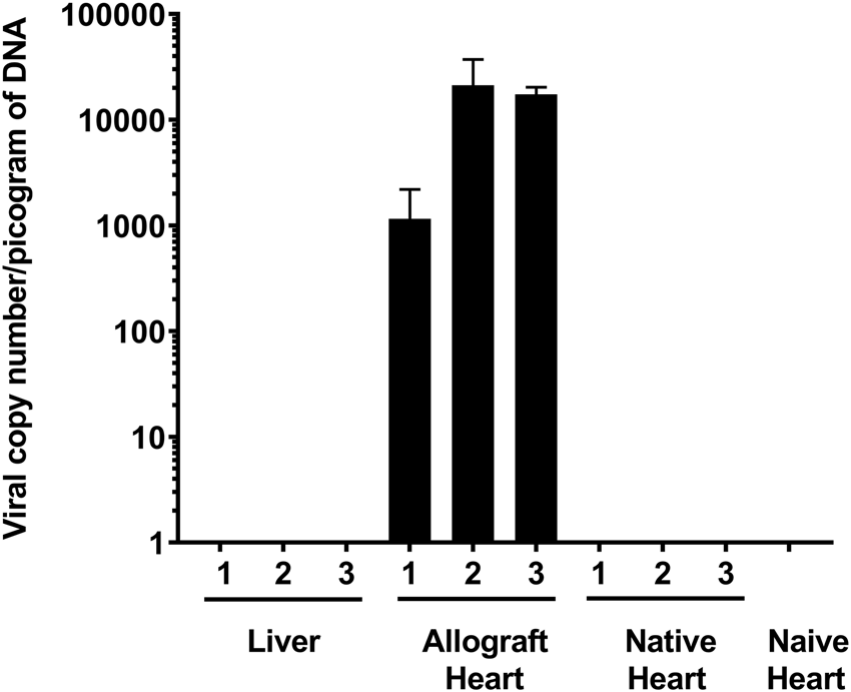
Quantitative real time PCR assessment of transgene copy number present per picogram of DNA isolated from liver, heart allograft, naïve and native hearts. (n = 3, except naïve heart n = 1).

Finally, the concentration of viral vector DNA in the allografts, native heart and livers of the recipient animals was examined by quantitative real time PCR using luciferase specific primers. High transgene copy number was confirmed in all three allografts, while recipient native heart and livers displayed no signal (Fig. 5). These findings of vector copy number mirror those of the luciferase gene expression and further supporting limited spread of vector from the transplanted organ.

## Discussion

Here we report that an *ex vivo*, normothermic perfusion system, currently in clinical trial for solid organ transplant storage and transport, can also be used as a platform to deliver a molecular therapy to the donor organ prior to implant. Unprecedented transgene expression was achieved in all regions of the allograft including the coronary vasculature. Despite strong expression in all regions of the allograft, transgene expression was not evident in any of the other organs from the recipient. Finally, the duration of perfusion (with the viral vector) for these experimental transplants is consistent with the duration of perfusion that is utilized for clinical transplants.

Preclinical and clinical data strongly support the importance of route of delivery for efficacy and safety of viral vector transduction of the heart^3^. Early clinical trials utilized surgical thoracotomy and direct viral vector injections into the myocardium^14^. This approach achieved significant local transgene expression but even with multiple injections, the majority of the myocardium for a human size heart cannot be affected. Additional delivery techniques included catheter-based delivery to the endocardium^15^. Perhaps most promising was vector delivery via repeated catheter based intracoronary injections, however, without full cardiac isolation on cardiopulmonary bypass, significant viral vector reaches secondary organs. In fact, a recent large-scale review of gene therapy clinical trials for cardiac disease concluded that present delivery approaches (intracoronary or intravenous administration) might not deliver sufficient amounts of the vector to the target tissue^16^. In our study, repeated circulation of the viral vector increased time for viral vector interaction with cell surface primary and secondary receptors resulting in diffuse robust expression of the transgene in the allograft.

Shah *et al*. reported first that viral vectors could be delivered to an explanted rat heart *ex vivo* during the preservation period, thus increasing the time during which the vector is present in the vasculature^4^. However, vectors given into the coronary circulation prior to cold static storage achieved limited transgene expression. Limited transgene expression in this model probably resulted from lack of metabolic activity required for vector attachment to receptors and cellular uptake, with washout of the vector occurring during warm reperfusion. In the current study, a pig heterotopic transplant model was utilized as previously described^12^. This model was used because the pig heart is large enough to be placed on the clinical perfusion device and the pig donor provides adequate blood volume to prime the circuit. These similarities between pig and human hearts should allow for rapid clinical adoption. In addition, the heterotopic transplant model does not require the graft to support the systemic circulation, which allowed us to study gene expression originating from the viral vector, even if graft dysfunction occurred.

The proprietary solution for perfusion storage (Transmedics) did not intefere with the ability of the recombinant adenoviral vector to transduce cells in culture. However, once whole blood was mixed with the perfusion solution, a significant drop in viral transduction was observed and upon further investigation was found related to elements within the plasma and serum. Neutralizing antibodies in the plasma and serum fractions of blood are known to limit the success of viral based gene delivery and are often exclusion criteria for clinical trials involving viral vectors^17^. The presence of pre-existing neutralizing antibodies in the pig provides an explanation for the observations in these experiments. To mitigate this issue, subsequent experiments used only the cellular fraction of centrifuged heparinized donor blood for the perfusate. Clinically, this blood centrifugation process, termed “Cell Saving”, is commonly used to repurpose the blood that is lost during surgery and auto-transfuse concentrated red blood cells. Here we suggest that this process should enable removal of antibodies (along with other serum proteins), thus generating a perfusate that better supports viral vector transduction. Once the blood is washed, it is important to re-establish the correct electrolyte balance and oncotic pressure (Table 1).

In these experiments, recombinant adenoviral vectors efficiently transduced a large cardiac allograft during a relative short period of *ex vivo* perfusion. Expression of the luciferase transgene was robust in all chambers of the heart; furthermore, the cardiac vasculature also heavily expressed the transgene. While it is difficult to fully compare given many differences in experimental setup, our degree of transgene expression appears to exceed that achieved with other experimental methods of cardiac gene delivery such as catheter directed intracoronary injection or direct myocardial injection. Finally, expression appears to be isolated to the allograft. If there was release of vector from the graft, other recipient organs such as the liver or spleen would be most likely to absorb the particles and show transgene expression. In the recipient’s organs, transgene expression as determined by the luminescent assay and western blotting is not detected. Together, these findings suggest that viral vector delivery of therapeutic transgenes during *ex vivo* perfusion may be an efficient and safe strategy to transduce large allografts. Future work should focus on delivering therapeutic genes, which may improve graft function or decrease the immune response to the transplanted organ. Importantly, while our study focused on the heart, this strategy can be applied to other organs where *ex vivo* perfusion is utilized including the lungs, liver and kidney. Further research is required to elucidate whether other methods of *ex vivo* organ maintenance such as cold storage with intermittent perfusion may also be used for viral vector delivery^18^.

The current study employed an Adenoviral vector that offers robust early-onset gene expression that later wanes over time. This vector type, which also has the benefit of a large DNA packaging capacity, would be optimal for treatments requiring short-term limited gene expression. This might be the case for genes aimed at increasing early inotrope of the heart during the early post-operative period, without the risk of arrhythmias, hypertrophy or heart failure associated with long term expression. The *ex vivo* perfusion approach should be applicable to utilization of other types of vectors (both viral and non-viral). We plan to next investigate vectors based on adeno associated virus (AAV) using the *ex vivo* perfusion approach as these types of vectors offer benefits of long-term gene expression in the allograft and less immunogenicity than Ad vectors^19^. AAV based vectors would be optimal for therapies using genes targeted at suppressing the immune response against the allograft where long-term, sustained gene expression would be desired. The effect of triple drug immunosuppression, which was used in these experimental transplants, on the transgene expression is not understood but may provide a substantial positive effect on duration of transgene expression. We did not perform a full histological analysis of immune cell infiltration into the allografts. This was mainly due to the overall short survival time but these experiments will be addressed in future work. Given the very high luciferase protein expression in the allograft as compared to any of the other organs in all three animals, we believe that three animals was a sufficient number to support the conclusions of this manuscript. The level of expression of the transgene (and also DNA copy number) in the allograft exceeds that detected in surrounding tissues by greater than many orders of magnitude. This extreme difference supports the low number of experiments. Furthermore, the overall cost and effort required to perform these large animal heart transplant experiments is far greater than what is required, for example, for rodent or *in vitro* studies of vector efficiency. Adding more animals to the study would provide low additional value. Future studies will examine therapeutic transgenes which will require a significantly increased number of animals to demonstrate therapeutic efficacy.

## Conclusion

Robust and diffuse transduction from an adenoviral vector can be achieved in the cardiac allograft using an *ex vivo* organ perfusion strategy to deliver the viral vector prior to transplantation. Notably, transgene expression was highly elevated in all parts of the allograft without detectable expression in any of the other organs of the recipient. This introduces a novel method of viral vector delivery that may be able to genetically modify organs prior to transplantation.
